# Postembryonic development and lifestyle shift in the commensal ribbon worm

**DOI:** 10.1186/s12983-024-00533-3

**Published:** 2024-05-06

**Authors:** Natsumi Hookabe, Rei Ueshima, Toru Miura

**Affiliations:** 1 Research Institute for Global Change (RIGC), Japan Agency for Marine-Earth Science and Technology (JAMSTEC), Yokosuka, Kanagawa 237-0061 Japan; 2 Department of Biological Sciences, Graduate School of Science, The University of Tokyo, Bunkyo, Tokyo 113-0033 Japan; 3 Misaki Marine Biological Station, School of Science, The University of Tokyo, Miura, Kanagawa 238-0225 Japan

**Keywords:** Lophotrochozoan, Marine, Phalloidin, Spiralia, Symbiosis, Tissue invagination

## Abstract

**Background:**

Various morphological adaptations are associated with symbiotic relationships between organisms. One such adaptation is seen in the nemertean genus *Malacobdella*. All species in the genus are commensals of molluscan hosts, attaching to the surface of host mantles with a terminal sucker. *Malacobdella* possesses several unique characteristics within the order Monostilifera, exhibiting the terminal sucker and the absence of eyes and apical/cerebral organs, which are related to their adaptation to a commensal lifestyle. Nevertheless, the developmental processes that give rise to these morphological characteristics during their transition from free-living larvae to commensal adults remain uncertain.

**Results:**

In the present study, therefore, we visualized the developmental processes of the internal morphologies during postembryonic larval stages using fluorescent molecular markers. We demonstrated the developmental processes, including the formation of the sucker primordium and the functional sucker. Furthermore, our data revealed that sensory organs, including apical/cerebral organs, formed in embryonic and early postembryonic stages but degenerated in the late postembryonic stage prior to settlement within their host using a terminal sucker.

**Conclusions:**

This study reveals the formation of the terminal sucker through tissue invagination, shedding light on its adhesion mechanism. Sucker muscle development likely originates from body wall muscles. Notably, *M. japonica* exhibits negative phototaxis despite lacking larval ocelli. This observation suggests a potential role for other sensory mechanisms, such as the apical and cerebral organs identified in the larvae, in facilitating settlement and adhesive behaviors. The loss of sensory organs during larval development might reflect a transition from planktonic feeding to a stable, host-associated lifestyle. This study also emphasizes the need for further studies to explore the phylogenetic relationships within the infraorder Amphiporiina and investigate the postembryonic development of neuromuscular systems in closely related taxa to gain a more comprehensive understanding of ecological adaptations in Nemertea.

## Background

Symbiosis, including parasitism, commensalism, and mutualism, is common in marine ecosystems [1–4]. Symbiotic associations bring about anatomical, physiological and/or reproductive modifications in symbiont species, thereby catalyzing the morphological and ecological diversification of many organisms [2, 5, 6]. In within-the-host-body commensal relationships, hosts provide a living space within their bodies for symbionts to reside for a part of or the entire duration of the symbionts’ lifespan [5]. Endosymbionts often undergo simplification of their body plan, including reductions in body size and appendages (e.g., [7, 8]), which are likely adaptations to their host environment. These modifications mostly occur during the postembryonic development of symbiont species. For example, in pea crabs that inhabit bivalves, postlarval development greatly differs from the typical pattern exhibited by free-living brachyurans [9]. Characterizing the ontogenetic process of symbiont species with modified body plans and understanding the deviation from the original development patterns in ancestral free-living species will be an approach to reveal such primary processes driving the diversification of body plans in relation to symbiotic associations.

Nemertea (ribbon worms), a phylum in Lophotrochozoa [10–12], encompasses soft-bodied worms characterized by having an eversible proboscis housed in a fluid-filled chamber, referred to as rhynchocoel [13]. It currently contains approximately 1350 species [14], mostly found in marine benthic habitats as free-living macrophagous carnivores on polychaetes, molluscs, and small crustaceans or scavengers [15–17]. As in other lophotrochozoan spiralian phyla, nemerteans exhibit stereotypical equal spiral cleavage [18–21], followed by gastrulation and larval development involving the formation of imaginal discs and organ rudiments [21, 22]. Nemertean larvae are classified into two types: planuliform and pilidium larvae [20, 21]. Of the approximately 50 known species of symbiotic nemerteans, the majority belong to Eumonostilifera (Hoplonemertea) with planuliform larvae. Their developmental processes are generally direct, and the transition from the embryo to juvenile does not involve a dramatic change in the body plan or a conspicuous metamorphosis [20].

**Fig. 1:**
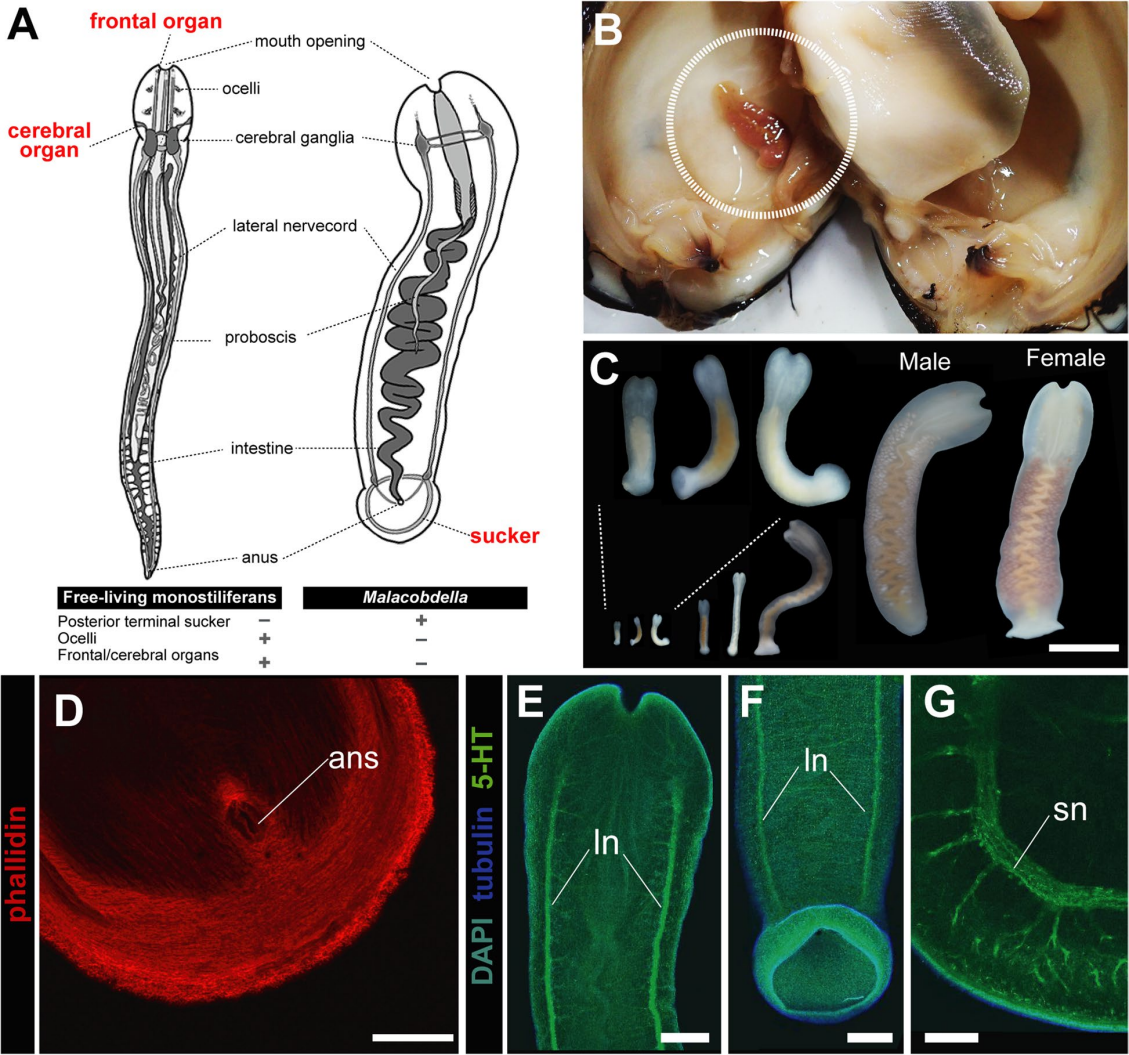
General morphology in the adult and juvenile stages of *Malacobdella japonica*. **A** A schematic image of free-living monostiliferan (left) and *Malacobdella* (right). **B** *M. japonica* inhabiting the mantle cavity of *Pseudocardium sachalinense*. **C** Juveniles and reproductive adults of *M. japonica*. **D** Muscles of the posterior sucker, juvenile. **E** The anterior nervous system of the juvenile. **F** The posterior nervous system of the juvenile. **G** A nervous ring of the posterior sucker, juvenile. Scale bars: 1 cm (**C**); 20 μm (**D**); 50 μm (**E**, **F**); 30 μm. Abbreviations: ans, anus; ln, lateral nerve cord; sn, sucker nerve

A developmental study on symbiotic monostiliferans is currently limited to the genus *Malacobdella* by Hammarstein (1918) [18]. Phylogenetically, the genus is nested within Eumonostilifera [23]. However, prior to the molecular phylogenetic studies, *Malacobdella* was considered a separate order, Bdellonemertea [24], due to its unique characteristics, including the absence of a stylet apparatus in the proboscis (Fig. 1A). *Malacobdella* species are characterized by having a flat body with a terminal sucker and lacking eyes and frontal and cerebral organs [25–27] (Fig. 1A). These morphological characteristics are considered to be established between the planktonic and host-settling periods. Using their terminal sucker, they settle and attach to the mantles of the host bivalves (Fig. 1A, B). Previous studies on the diet of *Malacobdella* species through examination of gut contents and stable isotope analyses have shown that they share similar trophic levels with their hosts, feeding on microalgae and diatoms [28]. Thus, *Malacobdella* species are generally considered to be kleptivores. Basically, there is only a single adult specimen per bivalve host.

Despite the remarkable ecological differences in their adults, *Malacobella* larvae exhibit no marked differences in morphological and ecological characteristics from other free-living monostiliferans, particularly during their planktonic larval stage (e.g., [18, 29]). In this study, we hypothesized that the unique morphology of *Malacobdella*, such as the terminal sucker and absence of eyes and apical/cerebral organs, forms during postembryonic development between the planktonic and host-settling periods in relation to their lifestyle shift from free-living larvae to commensal within bivalve mantles. Given that larval ocelli and apical/cerebral organs are important for searching and locating their host, these sensory organs might have once formed in their postembryonic stage prior to functioning of their posterior sucker for settlement. Since the developmental process of *Malacobdella* has been poorly understood, with the only available example being a descriptive developmental study on *M. grossa* in Hammarstein’s (1918) dissertation [18], this study first clarified the embryonic and postembryonic development of the genus. In the present study, we observed embryonic and postembryonic development in *M. japonica*, with a specific focus on unique characteristics of the genus: (*i*) terminal sucker, (*ii*) ocelli, and (*iii*) apical/cerebral organs. The data obtained were then compared with existing descriptions of larval development in other monostiliferous nemerteans.

## Results

### Species identification

Species identification of the Akkeshi specimens used in the present study (Fig. 1B, C) was performed through COI barcoding. The 566 bp COI sequences obtained from the Akkeshi specimens were found to be an exact match with those of specimens collected from the same host species at Kashima-nada, situated approximately 75 km from Kujukuri (type locality), thereby confirming their identification as *M. japonica*.

### Early development in M. Japonica

**Fig. 2:**
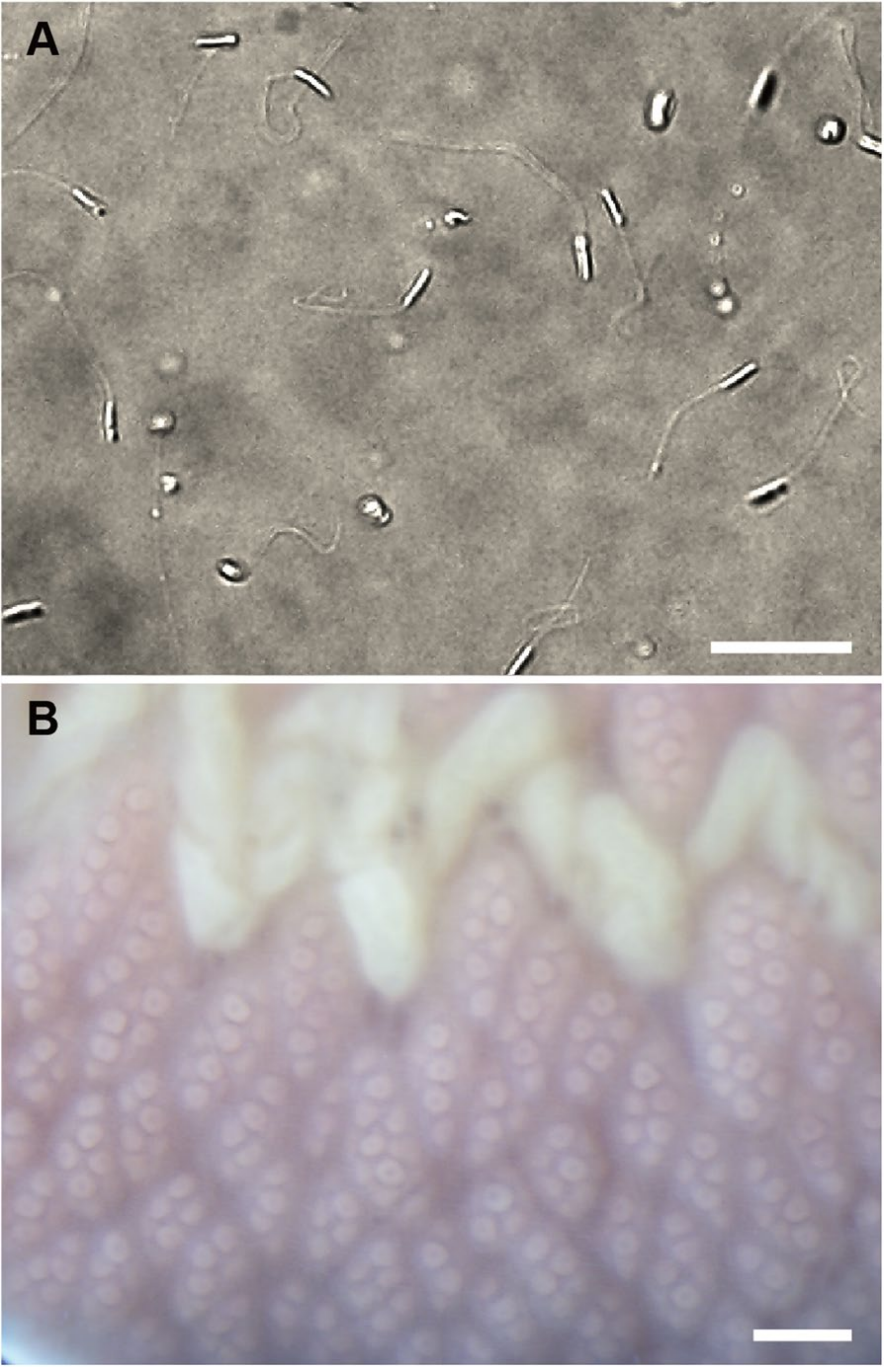
Gametes of *M. japonica*. **A** Sperm. **B** Oocytes. Scales: 10 μm (**A**); 500 μm (**B**)

In *M. japonica*, gravid females possess uniformly pale pinkish ovaries (Figs. 1C and 2B). Oocytes dissected from the female body were first triangular but subsequently became round, undergoing geminal vesicle breakdown within 1–6 h after exposure to FSW. Oocytes naturally spawned were round, 182.6 μm in diameter, surrounded by an egg chorion 347.5 μm in diameter and a transparent layer of jerry 670.5 μm in diameter on average (*N* = 5). Mature males are pale-colored due to the metamerically arranged testes (Fig. 1C). The sperm head is filiform and 6.5 μm in length on average (*N* = 6) (Fig. 2A).

**Fig. 3:**
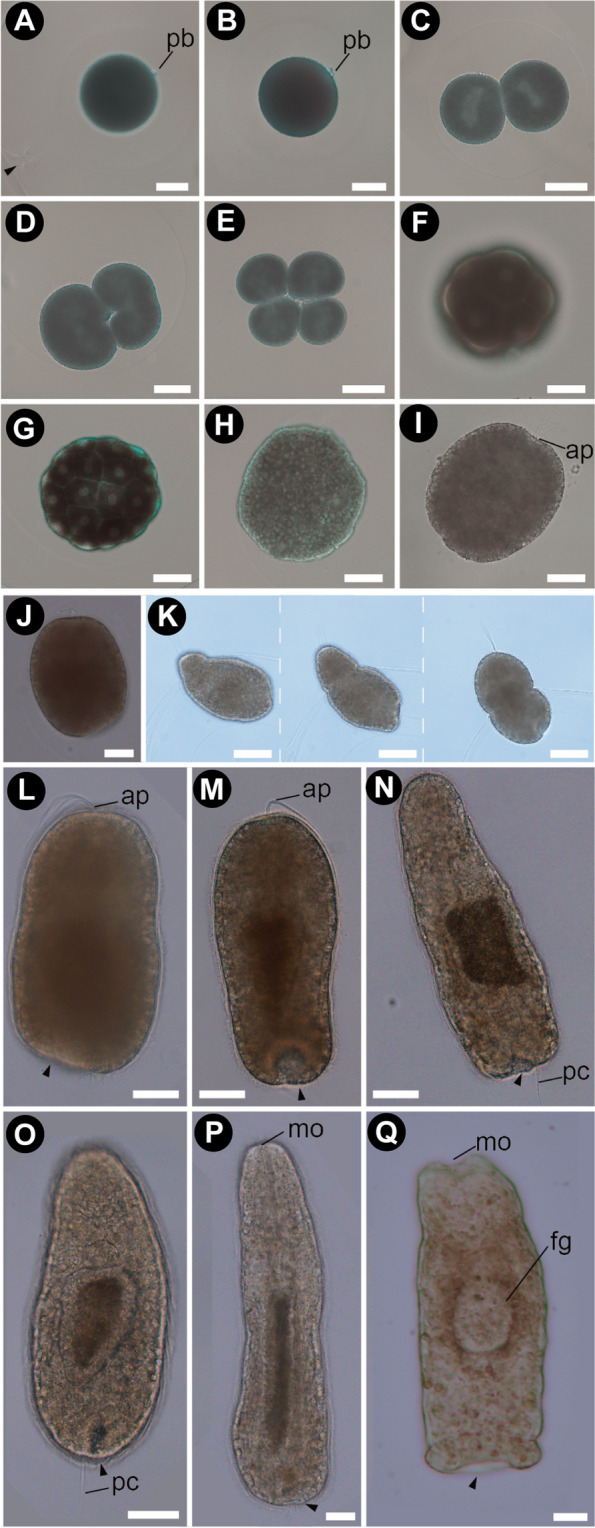
Early development of *M. japonica*. **A** Fertilized egg forming the first polar body. **B** Formation of the second polar body. **C** First cleavage. **D** Second cleavage furrow. **E** The 4-cell stage, polar view. **F** The 8-cell stage, lateral view. **G** The 32-cell stage, lateral view. **H** Morula stage. **I** Prehatching stage, rotating in the egg chorion with an apical tuft and cilia. **J** 72-hour-old embryo with the posterior end fading. **K** 84-hour-old larva hatching from the egg chorion. **L** 84-hour-old larva with a prominent apical tuft, anteroposteriorly becoming elongated. The posterior region appeared lighter colored. **M** 4-day-old larva showing a conspicuous posterior invagination. **N** 5-day-old larva with a posterior cirrus. **O** 7-day-old larva showing the foregut. **P** 14-day-old larva showing the mouth opening and anteroposteriorly elongated foregut. **Q** 21-day-old larva with the intestine no longer filled with yolk granules. In this stage, posterior invagination resulted in a distinct sucker-like structure in the ventroposterior end. The arrowheads point to the posterior invagination. Scales: 50 μm (**A**–**H**); 80 μm (**I**–**Q**). Abbreviations: ap, apical tuft; fg, foregut; mo, mouth; pb, polar body; pc, posterior cirrus

Once fertilized, spiral holoblastic cleavage was performed in laboratory culture (Fig. 3). At 15 °C, the first polar body is formed 30–60 min after fertilization on the opposite side of sperm penetration (Fig. 3A). The second polar body is formed 60–90 min after fertilization (Fig. 3B). The first cleavage was observed at approximately 2 h after fertilization (Fig. 3C). Blastomeres at the 2-cell stage began the subsequent cleavage 2 h 30 min–3 h after fertilization (Fig. 3D), resulting in 4-cell embryos (Fig. 3E). Further cell divisions were observed approximately every 30 min (Fig. 3F–H); 32-cell embryos were observed within 4.5 h after fertilization.

**Fig. 4:**
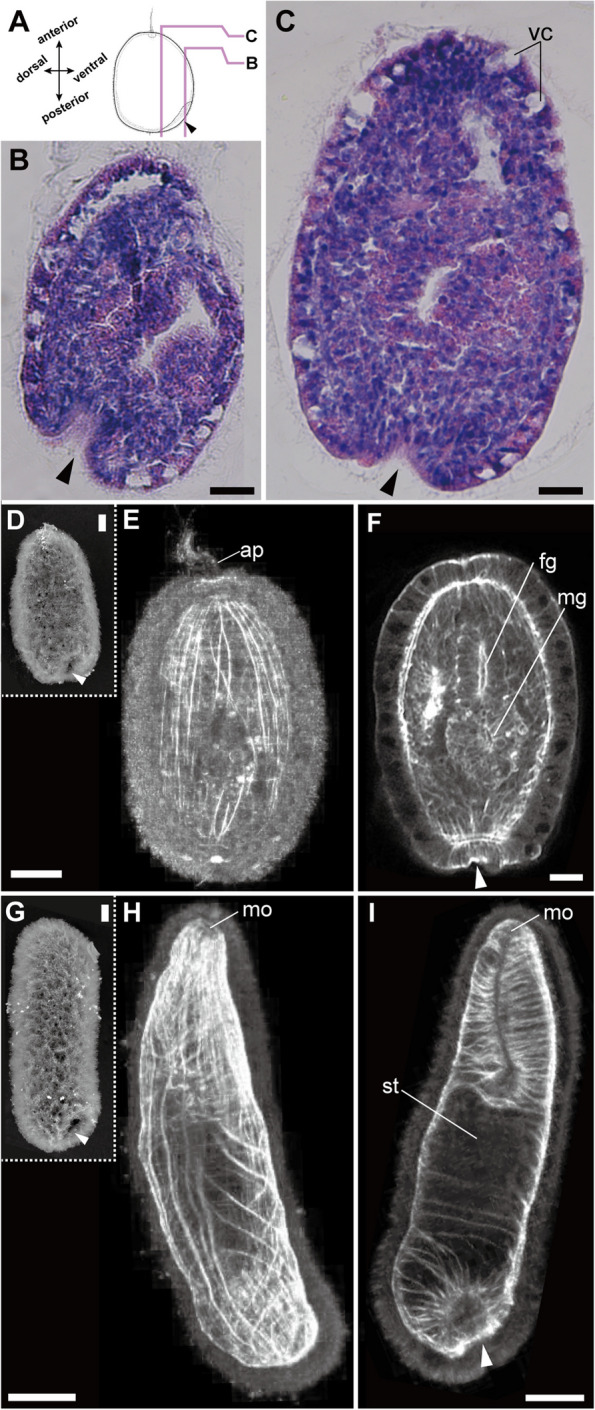
Posterior invagination in 72-hour embryos (**A**–**C**, **D**, **E**, **G**, **H**) and 14-day-old larvae (**G**–**I**). **A** Schematic image showing the locations of histological sections and HE staining. **B** A horizontal section at the ventral side. **C** A horizontal section across the apical tuft. The arrowheads point to the posterior invagination. **D** 72-hour-old larvae, SEM. **E** 72-hour-old larvae, a substack of a frontal section, showing well-developed body wall muscles labeled with phalloidin. **F** A 72-hour-old larva, a substack of a frontal section, showing the internal organs, including the foregut (fg) and midgut (mg). **G** 14-day-old larvae, SEM. **H** Fourteen-day-old larvae, a substack of a frontal section, showing well-developed diagonal muscles of the body wall labeled with phalloidin. **I** Fourteen-day-old larvae, a substack of a frontal section, showing the mouth opening leading into the foregut lumen. The arrowheads point to the posterior invagination. Scales: 50 μm (**B**, **C**, **E**, **F**); 80 μm (**H**, **I**). Abbreviations: ap, apical tuft; fg, foregut; mg, midgut; mo, mouth opening; st, stomach; vc, vacuolated cells in epidermis

**Fig. 5:**
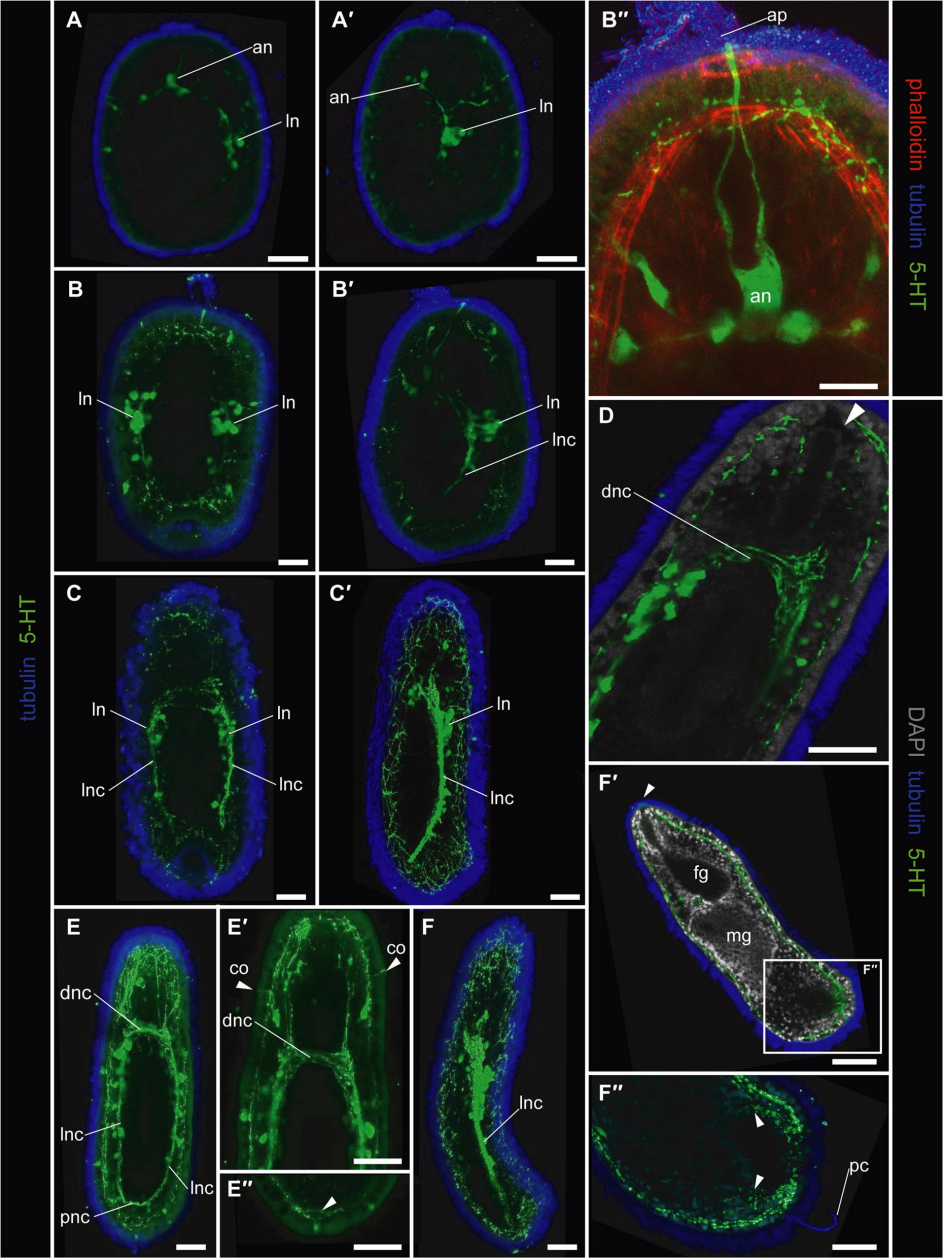
Development of the serotonergic nervous system of *M. japonica.* Green: 5-HT-like immunoreactivity; blue or purple (B”): cilia, acetylated tubulin immunoreactivity; cyan: nuclei labeled with DAPI; red: muscles labeled with phalloidin. (A, A’) 48-hour-old larvae, a substack from dorsal view (A) and a substack lateral view (A’) showing apical neurons (an) leading to lateral neurons (ln) via lateral nerve cords. (B, B’, B”); 72-hour-old larvae, a substack from dorsal view (B), a substack from lateral view (B’), and a substack from dorsal view with higher magnification of apical neurons (B’). (C, C’) 5-day-old larvae, a substack from dorsal view (C) and a substack lateral view (C’) showing well-developed lateral nerve cords. (D) 7-day-old larvae with higher magnification of dorsal nerve commissure (dnc), an arrowhead pointing to the mouth opening. (E, E’, E”) 14-day-old larvae, a substack from dorsal view (E), a substack from dorsal view, with higher magnification of dorsal nerve commissure (dnc) and nerve processes of 5-HT-like immunoreactive cells running from cephalic nerves (cn) into the body wall (arrowheads pointing to cerebral organs) (E’) (E”), and magnification of posterior nerve ring pointed with an arrowhead, a substack from dorsal view (E”). (F, F’) 21-day-old larvae, a substack from lateral view (F) and a substack from ventral view, mouth opening (arrowhead) leading to foregut lumen (F’). Scale bars: 30 μm. Abbreviations: an, apical neuron; ap, apical tuft; co, cerebral organ; dnc, dorsal nerve commissure; fg, foregut; ln, lateral neuron; lnc, lateral nerve cord; mg, midgut; pc, posterior cirrus; pnc, posterior nerve commissure

After the morula stage (Fig. 3H, I), embryos were ciliated and slightly elongated in the anteroposterior axis as early as 48 h after fertilization. At this stage, the epithelium in the posterior region became rough, partially lacking cilia (Fig. 3I). By 66 h after fertilization, embryos possessing an apical tuft rotated inside the egg chorion. The posterior end of the 66-hour-old embryo became flattened. At this stage, blastopore-like depressions in the vegetal pole were not distinguished through observation with a light microscope. By 72 h after fertilization (Fig. 3J), posterior invagination occurred in the middle of the flattened posterior end, as observed with histological sections (Fig. 4A–C) and SEM (Fig. 4D). Larvae with an apical tuft hatched at 84 h after fertilization (Fig. 3K) and swam with uniformly distributed cilia (Fig. 3L). During all prior periods, the posterior invagination was confirmed as a lighter region in the posterior end (Fig. 3L and M, pointed with an arrowhead). A posterior cirrus originated from the dorsal side of posterior invagination at 5 days after fertilization (Fig. 3N). Once hatched from the egg chorion, the larval body anteroposteriorly elongated, developing a pouch-like stomach within 7 days after fertilization (Fig. 3O). The mouth opening was confirmed as early as 14 days after fertilization (Fig. 3P). As early as 14 days after fertilization, larvae began to crawl on the bottom of a plastic container. Within 21 days after fertilization, the larva appeared to settle on the bottom of a plastic container with a terminal sucker (Fig. 3Q). At 7–21 days after fertilization, internal organs, including alimentary canal (foregut, midgut, and intestine) and nervous system elements (cerebral ganglia, cerebral organs, and lateral nerves), developed (Figs. 4 and 5).

### Development of musculature and digestive systems during postembryonic development

The body-wall musculature of *M. japonica* was composed of outer circular, middle diagonal, and inner longitudinal muscle layers in juveniles (Fig. 1D). Longitudinal and circular muscles of the body wall were detected in the 72-hour-old embryo (Fig. 4E). Circular muscles densely developed at the posterior end of the body wall (Fig. 4F). In addition to longitudinal and circular muscles, diagonal muscles developed in the 14-day-old larvae (Fig. 4H). Splanchnic muscles well developed from the mouth opening to the foregut region (Fig. 4I).

The gut lumen became distinct 72 hours after fertilization (Fig. 4F). In this stage, the foregut appeared to be unconnected to the midgut (Fig. 4F). Within 14 days after fertilization, the foregut was anteriorly elongated to form the mouth opening (Fig. 4I). In the 14-day-old larvae, the stomach and foregut were still partitioned by several transverse muscle fibers (Fig. 4I). In the 21-day-old larva, the foregut was further enlarged and finally connected to the midgut (Fig. 5F’).

### Sucker formation at the posterior end

In the prehatching 72-hour-old embryo, an epithelial invagination was observed on the ventral side of the posterior cirrus (Figs. 3J and 4D). The larval epidermis in this stage contains multiciliated cells, mucous cells, and vacuolated cells (Fig. 4A–C). Vacuolated cells were not observed around the posterior invagination (Fig. 4B, C and F). During the period 3–4 days after fertilization, the posterior invagination is distinguishable under a light microscope from other epidermal tissue due to the lighter coloration (Fig. 3J). Within 5 days after fertilization, the posterior invagination became more conspicuous and more deeply depressed (Fig. 3M, N). The posterior depression sometimes contracts due to muscle contraction when swimming or crawling 7–21 days after fertilization (Figs. 3O, P and 5B). In the rim of the posterior invagination (Fig. 4G), radial muscle fibers developed in the 14-day-old larvae (Fig. 4I). After settling to the bottom of a plastic container, 21 days after fertilization, the posterior epithelial tissues surrounding the posterior depression became thicker and resulted in a sucker-like structure (Fig. 3Q). Once the settled larva was exposed to water current produced by a plastic pipette, they were attached to the bottom with the posterior sucker.

### Development of the nervous system, including ocelli and apical/cerebral organs

In the juveniles of *M. japonica*, apical/cerebral organs were not detected (Fig. 1E, F) as in their adults (Fig. 1A). Lateral nerve cords were well labeled with anti-5-HT antibodies (Fig. 1E, F), posteriorly innervating the sucker nerve (Fig. 1G). A ring of the sucker nerve peripherally innervates the nerve plexus, branching into a more diffuse nerve fiber (Fig. 1G).

5-HT-like immunoreactive components were first detectable under cLSM earlier than hatching from the egg chorion 48 h after fertilization (Fig. 5A). Neurons in this stage comprised apical and paired lateral neurons; the two were connected by neurites (Fig. 5A, A’). An apical organ was confirmed as an apical plate innervated by paired apical neurites (Fig. 5A’); the innervations run through muscular rings labeled with phalloidin (Fig. 5B’). In the 72-hour-old larvae after hatching from the egg chorion, two neurites originating from apical neurons fused to each other between the epithelium and muscle layers, anteriorly extending to the apical tuft (Fig. 5B”). Subapical neurons developed on both lateral sides of the apical neurons (Fig. 5B”). In this stage, the number of lateral neurons was increased (Fig. 5B, B’). The nervous commissure of lateral nerve cords became slightly visible in the 5-day-old larvae (Fig. 5C) and turned out to be conspicuous in the 7-day-old larvae, forming the dorsal nerve commissure (Fig. 5D). During these periods, apical neurons became invisible as 5-HT immunoreactive components (Fig. 5C, C’, D). The lateral nerve cords also joined each other with the posterior nerve commissure in the 14-day larvae (Fig. 5E). At the higher magnification of the stage, a pair of neurons precerebrally were innervated from the dorsal nerve commissure (Fig. 5E’). Neurites originating from the paired precerebral neurons extended to the epithelium (Fig. 5E’). At the posterior end of the 14-day-old larvae, the subepithelial nerve plexus formed a ring to rim the posterior invagination (Fig. 5E”). The serotonergic nervous system became more complex 21 days after fertilization with paired lateral nerve cords with well-developed lateral neurons (Fig. 5F). In the rim of the posterior invagination on the ventral side of the posterior cirrus, the subepithelial nerve plexus developed incompletely encircling the posterior invagination in this stage (Fig. 5F”).

**Fig. 6:**
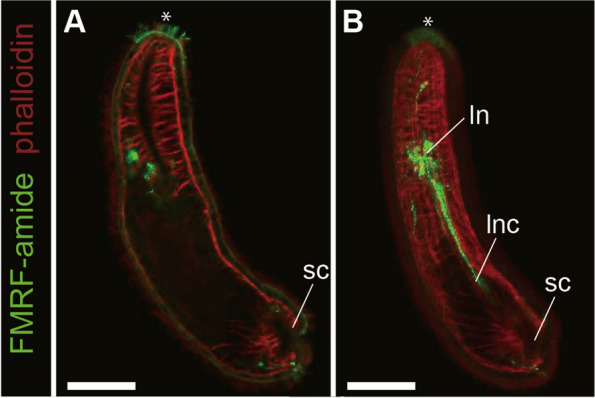
Localization of FMRF-amide immunoreactive cells in the nervous system of 14-day-old *M. japonica* larvae. Green: FMRF-amide-like immunoreactivity; red: muscles labeled with phalloidin. **A** A substack from dorsal view, an asterisk indicating FMRF-amide reactive cells densely located at the anterior tip. **B** A substack from lateral view, showing lateral neurons, lateral nerve cords, and FMRF-amide reactive cells in the anterior tip indicated with an asterisk. Abbreviations: ln, lateral neuron; lnc, lateral nerve cord; sc, sucker primordium

Additionally, FMRF-amide signals were examined in the 14-day-old larvae of *M. japonica.* FMRF-amide-like immunoreactive components were found at the anterior tip of the head (Fig. 6A) and the paired lateral neurons (Fig. 6B).

## Discussion

### Comparison to free-living monostiliferans

In *M. japonica*, they undergo direct development without significant changes in body structure throughout embryonic and larval stages, similar to other previously studied free-living monostiliferans (e.g [20, 30]). However, there were four major differences in the development of internal organs between *M. japonica* and other monostiliferans. One difference is that the formation of the proboscis and the stylet apparatus was not observed in *M. japonica*, at least within one month after fertilization. This suggests that planktonic larvae of *M. japonica* are lecithotrophic at least during the period unlike a predatory planktotrophic phase in *Emplectonemma* [30]. The reduced proboscis of *Malacobdella* adults may form after host settling, while the stylet is completely lost throughout its life stages. Three other notable differences were the formation of the *i*) sucker primordium, *ii*) ocelli, and *iii*) apical/cerebral organs (further details are discussed below).

### Posterior invagination and sucker formation in *M. japonica*

In the present study, we demonstrated that the terminal sucker formed through the flattening and subsequent inward bending of the posterior epithelium on the ventral side of the posterior cirrus (Fig. 4D–I). The morphogenesis of the posterior sucker in *M. japonica* appeared similar to so-called “tissue invagination”, which is one of the fundamental mechanisms in the early development of metazoan body plans [31, 32] and is commonly observed during gastrulation in invertebrates [31–33], including nemerteans [21, 34]. Although the existence of a sucker primordium has been shown in Hammarstein’s illustrations ([18], Figs. 14, 15, 16, 17 and 18), the sequential process of the development stages from the sucker primordium to the functional sucker is first reported in the present study.

Suckers, as adhesive organs, evolved independently in several lophotrochozoan taxa, such as leeches, branchiobdellids (Annelida), cephalopods (Mollusca), and parasitic/free-living flatworms (Platyhelminthes) [35]. However, detailed morphological studies on the formation of suckers during embryonic and postembryonic development are largely limited to cephalopods (e.g., [36–38]). These suckers initially form as buds composed of a single layer of epithelial cells, which subsequently invaginate toward the oral side to form a furrow [38]. The sucker formation process involving epithelial tissue invagination seems to commonly occur at the onset of sucker formation in both cephalopods and nemerteans. In having a simple, cup-like form, the sucker of *Malacobdella* resembles a terminal sucker of leeches or parasitic/free-living flatworms in Rhabdocoela, such as Temnocephalidae Monticelli, 1899 [39], rather than cephalopods. However, the detailed processes of sucker formation in lophotrochozoan taxa remain uncertain, so further explorations into platyhelminths and leeches are required to reveal whether the sucker formation processes evolved convergently in Lophotrochozoa.

**Fig. 7:**
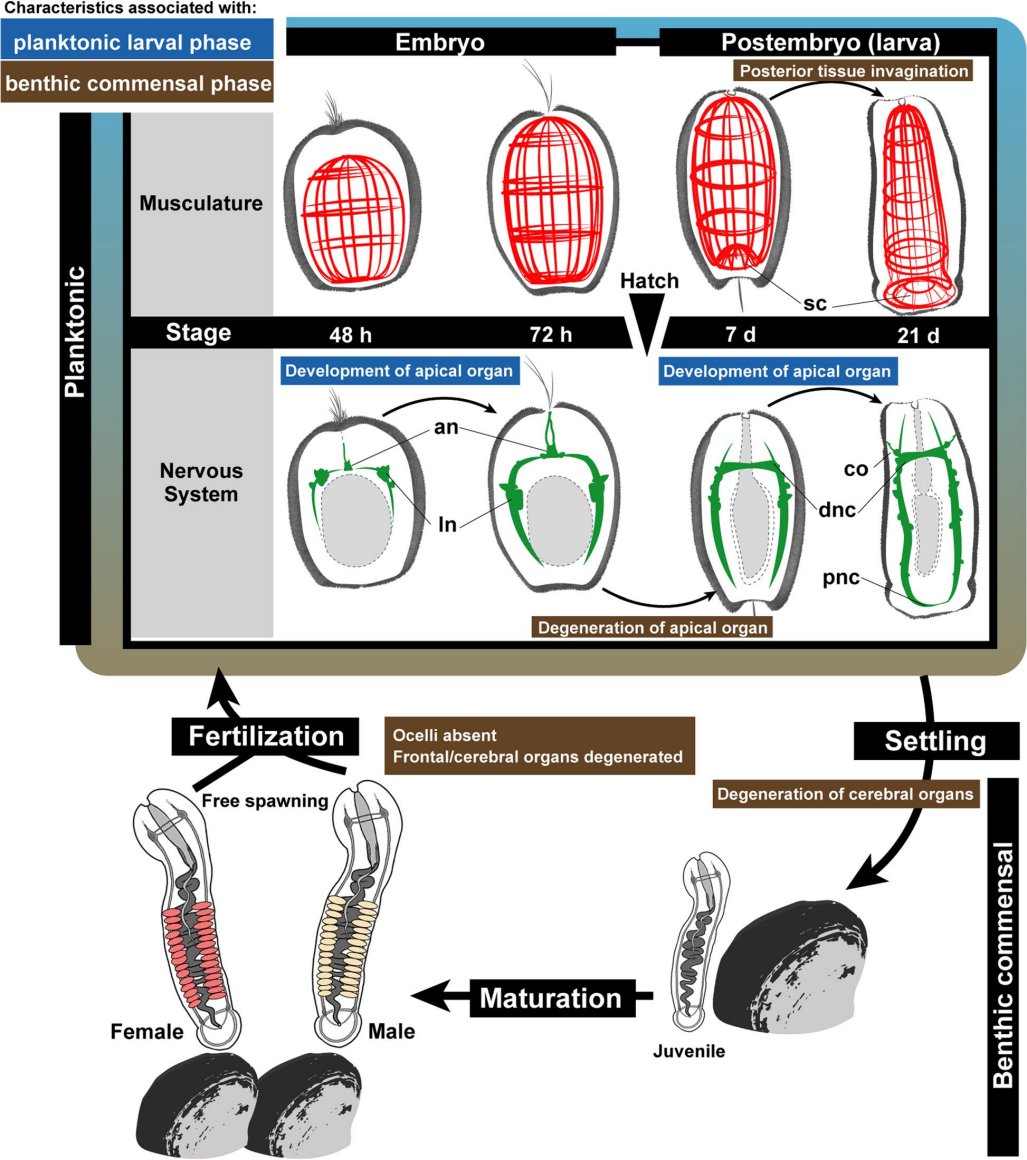
Schematic summary of the embryonic/postembryonic development of *M. japonica* at 15 °C and their life cycle. Abbreviations: an, apical neuron; co, cerebral organ; dnc, dorsal nerve commissure; ln, lateral neuron; pnc, posterior nerve commissure; sc, sucker primordium

Our findings also infer the underlying mechanism of sucker muscle formation. The sucker muscles of *Malacobdella* are comprised of longitudinal, circular, and radial muscles (Fig. 1D), similar to the terminal sucker of leeches [40, 41]. By coordinating the contractions of these muscles in various orientations, the sucker is able to generate a negative pressure for adhesion [40]. The sucker muscular system is important for adhesion by suction, yet the formation process is poorly understood. Based on our morphological data, we presumed that the sucker muscular system might originate from body wall longitudinal and circular muscles (Fig. 7). Through the simple process of posterior tissue invagination, where the posterior tissue is bent inward and the folded parts of the posterior tissue are flattened, the circular muscles of the body wall appear to give rise to the circular muscles of the sucker, with the longitudinal muscles of the body wall to the longitudinal and radial muscles of the sucker (Fig. 7).

The nervous system of the sucker seems to be characterized by a nerve ring running along the rim of the sucker plate (e.g., [42]). In the present study, we observed the nerve ring in juvenile specimens (Fig. 1G), which is not known in other monostiliferans, but the formation process was incompletely observed during the postembryonic larval stage (Fig. 5F”). This suggests that development of the nervous system in the posterior sucker may be complete after the transition from swimming to settling stages (Fig. 7).

The sucker of *Malacobdella* represents an evolutionary novel character within the phylum Nemertea, likely arising as an adaptation to their endocommensal lifestyle in the mantle cavity of bivalves. While other members of the phylum, such as the monostiliferan *Tetrastemma fozoensis* Junoy, 2008 from *Scrobicularia plana* and the heteronemertean *Uchidana parasita* Iwata, 1967 *Mactra sulcataria*, are known to inhabit the mantle cavities of bivalves, none possess a terminal adhesive organ.

### Absence of ocelli in the larvae of *M. japonica*

Our study on the larval development of *M. japonica* provides new insight into ocelli development in the taxon. We did not observe larval ocelli with black pigments during ontogeny in *M. japonica*, and it was not confirmed as immunoreactive signals for 5-HT and FMRF-amide. This result suggests that there are at least two distinct patterns for ocelli development in *Malacobdella*. One pattern, reported in *M. arrokeana* and *M. grossa*, is that ocelli developed during the swimming larval stage but were lost in the juvenile or adult stages, probably because photoreception would not be necessary for their endocommensal lifestyle. In contrast, ocelli appear to be suppressed even in the larval stage in *M. japonica*.

In general, simple ocelli in planktonic larvae of marine invertebrates mediate phototaxis, allowing the larvae to swim freely and eventually settle on bottom substrates or host animals [43]. Therefore, the presence or absence of larval ocelli is considered to be related to their behavior toward light stimuli [43, 44]. In the present study, negative phototaxis was observed in the larvae of *M. japonica* (Hookabe, personal observation), as reported in *M. arrokeana* [29], despite the absence of ocelli in the larvae of *M. japonica.* Negative phototaxis in *Malacobdella* larvae appears to enable them to settle on host bivalves buried in sandy to muddy substrates, but it might not be regulated by photosensors in the ocelli.

### Apical and cerebral organs ontogenetically lost in *Malacobdella*

The frontal and cerebral organs are major sensory receptors in the head of ribbon worms that detect physiological and chemical stimulation in the water, allowing them to efficiently track and catch prey [27, 45–47]. In free-living monostiliferous nemerteans, the frontal organs develop from larval apical organs in the early larval stage, while the cerebral organs appear in the middle to late stages of larval development [48, 49]. Adults in *Malacobdella* lack frontal and cerebral organs [27, 50, 51]. The present study, however, revealed that larvae possessed both apical (Fig. 5B”) and cerebral organs (Fig. 5E). This suggests that the sensory organs developed but become reduced/lost during larval development (Fig. 7), probably due to the nonselective plankton-feeding habits of adults and their stable, host-associated habitat within the mantle cavity of bivalves [15].

Although not well understood, the function of cerebral organs in monostiliferous nemerteans is considered to be related to prey capture even during the planktonic period [52]. Therefore, it is reasonable that the cerebral organs in *M. japonica* develop during larval development (Fig. 5E’) for active feeding. Although the larvae of *M. japonica* are primarily lecithotrophic, it was observed that the yolk appeared to be already digested by the time the cerebral organs began to develop. The findings gave us insight into the ontogenetic loss of cerebral organs in *Malacobdella*; the sensory organs of *Malacobdella* developed during the planktonic larval phase, likely for effective prey capturing and/or sensing and locating hosts but are subsequently lost prior to the endocommensal phase (Fig. 7).

### Morphological evolution in postembryonic development in Nemertea

This study focuses on the morphological evolution of endosymbiotic ribbon worm showing adaptation to endocommensal lifestyles. In *Malacobdella*, unique morphological characteristics, such as the presence of a terminal sucker and the loss of sensory organs (apical/cerebral organs), formed in postembryonic stages. This finding aligns with a recent comparative study on embryonic/postembryonic development in Nemertea, which suggests that most of the within-clade morphological diversity in the phylum has resulted from postembryonic development [53]. Molecular phylogenetic analyses have shown that *Malacobdella* is robustly nested within the Amphiporiina and forms a clade with the land-dwelling monostiliferans *Geonemertes*, although the relationship is not well supported. Future studies should establish a robust phylogeny for the infraorder Amphiporiina within the order Monostilifera and investigate the postembryonic development of neuromuscular systems in taxa closely related to *Malacobdella*.

## Conclusions

This study provides insights into the postembryonic development and unique morphological adaptations of the nemertean genus *Malacobdella*, which has evolved a commensal lifestyle with molluscan hosts. Key findings include the revelation that the formation of the terminal sucker in *Malacobdella* involves tissue invagination, a fundamental mechanism found in various metazoans. Additionally, the loss of sensory organs during larval development reflects the transition from planktonic feeding to a stable, host-associated lifestyle within bivalve mantles in the genus. These findings contribute significantly to our understanding of the development and evolution of commensal organisms represented by *Malacobdella*. They also underscore the importance of future research into phylogenetic relationships within the infraorder Amphiporiina and the postembryonic development of neuromuscular systems in closely related taxa, promising a comprehensive understanding of these ecological adaptations within Nemertea.

## Methods

The individuals in this study are categorized as follows: adults, juveniles, embryos, and larvae. Adults are fully matured specimens with developed gonads obtained from bivalves. Juveniles are immature specimens obtained from bivalves. Embryos and larvae were obtained by artificial fertilization of captive adults; larvae were in stages after hatching from the egg chorion (84 h after fertilization in this study). These terms are used consistently throughout the study to refer to the respective categories of individuals.

### Collecting juveniles/adults and species identification

We collected juvenile/adult specimens from *Spisula sachalinensis* (Schrenck, 1862) obtained at a local marketplace in Akkeshi, Hokkaido, Japan. From the Far Eastern Seas, including the Sea of Okhotsk and the northwestern Pacific Ocean, two species of *Malacobdella* have been reported [54]: *M. grossa* (Müller, 1776), found in *Mya uzenensis* Nomura & Zinbo, 1937 [55], and *M. japonica* Takakura, 1897 (Fig. 1B, C), found in *S. sachalinensis* (Schrenck, 1862). To confirm the species identity of the specimens collected from Akkeshi, Hokkaido, Japan, that were used in this study, we compared partial sequences of cytochrome *c* oxidase subunit I (COI) with topotype specimens collected off Kashima-nada.

DNA extraction was performed using DNeasy Blood & Tissue Kits (Qiagen, Hilden). Partial sequences were amplified by polymerase chain reaction (PCR) using an Applied Systems 2720 thermal cycler with the primer pair LCO1490/HCO2198 [56]. The PCR protocol was as follows: preheating at 94 °C for 2 min; 35 cycles of 94 °C for 40 s, 50–52 °C for 75 s, and 72 °C for 60 s; and a final extension at 72 °C for 7 min. Sequencing was carried out using the same primer pairs and outsourced by AZENTA/GENEWIZ (Tokyo, Japan). Sequences newly obtained and deposited in GenBank under accession numbers OR592591 (Akkeshi) and OR592590 (Kashima) were aligned using CLUSTALW implemented in MEGAX ver. 10.2.4 [57].

### Obtaining gametes and embryonic cultures

Reproductive adults of *M. japonica* were carefully picked up from the host using a plastic pipette. In the laboratory, each specimen of *M. japonica* was kept alive in approximately 130 ml of filtered seawater (FSW) in a small plastic container (80 mm×80 mm×104 mm) without aeration. They were maintained in an incubator kept at 15 °C in a wine cellar equipped with a Zero Class SB-38 air conditioner (Sakura Inc., Tokyo); the water temperature was the average seawater temperature in Akkeshi during the study period. The water in the containers was changed two to three times weekly. Female gametes render the specimen of *M. japonica* reddish, while male gametes are pale to pinkish colored. Oocytes were obtained from occasionally spawning females or otherwise by dissection or puncturing the body surface with a needle. Naturally spawned or dissected oocytes were exposed to seawater for at least 2 h, which allowed primary oocytes to undergo germinal vesicle breakdown. Sperm were obtained by puncturing the body surface of the male specimen with a needle and then suspended with FSW. A drop of sperm suspension was added to approximately 400 ml of egg suspension with FSW in a plastic container (100 mm×100 mm×50 mm). Once the first polar body was observed, embryos were washed three times with FSW to remove excess sperm and to avoid bacterial infection.

Developing embryos were cultured in approximately 400 ml of FSW at 15 °C. Water was changed every day during the first week and every three days during the remaining study period. To prevent bacterial growth in embryonic cultures, dead larvae and empty egg chorions were removed.

Embryonic and larval development of *Malacobdella japonica* was observed in vitro from July to September 2020 and 2021. Juvenile specimens of *M. japonica* (Fig. 1C) were frequently found from March to April in the mantle cavity of *P. sachalinense.*

### Light microscopy

Developing embryos were carefully picked up, placed on a glass slide, and covered by a coverslip with spacers (0.1 mm-thick adhesive tape). Photographs were taken through an Olympus BX51 compound light microscope (Olympus, Tokyo) with a Nomarski differential interference contrast (DIC) device.

### Fluorescence staining and confocal laser scanning microscopy (cLSM)

Before fixation, embryos, larvae, and juveniles were anesthetized in MgCl_2_ solution (1:1 mixture of 7.5% MgCl_2_ and FSW) for 15 min at 15 °C. Embryos and larvae developed 2, 3, 5, 7, 14, and 21 days after fertilization were fixed in 4% paraformaldehyde at 4 °C for 15 min, while juveniles were fixed at 4 °C overnight. Fixed specimens were rinsed three times, washed for 15 min three times and then stored in phosphate-buffered saline (PBS) at 4 °C. Chorions were removed using shape forceps and a needle from the embryos at the earlier stages (48 and 72 h after fertilization). To permeabilize cell membranes, specimens were rinsed three times and washed for 15 min three times in PBS with 0.1% Triton X-100 (Nacarai Tesque, Inc., Kyoto) (PBT). The specimens were washed in PBT containing 1% bovine serum albumin (BBT) for 15 min three times and blocked in BBT with 2% normal goat serum for 30 min.

To visualize perceptive organs (ocelli and apical/cerebral organs) and their neurons in relation to their neuromuscular system, the specimens were double-stained using the following primary antibodies against acetylated α-tubulin (#T6793, Sigma-Aldrich (St. Louis), final concentration 1:600) and serotonin (5-hydroxytryptamine (5-HT) (#20,080, ImmunoStar (Wisconsin), final concentration 1:600) or FMRF-amide (#20,091, ImmunoStar, final concentration 1:600) for 2 h at room temperature or overnight at 4°C; since this study is the first to visualize the internal morphology of Malacobdellidae by immunohistochemistry, the three antibodies listed above were tested. After washing in BBT for 15 min three times, the larvae were incubated in BBT with secondary antibodies: Alexa Fluor 647 goat-anti-mouse secondary antibody (Thermo Fisher Scientific, Massachusetts) for acetylated α-tubulin and Alexa Fluor 488 donkey-anti-rabbit secondary antibody (Thermo Fisher Scientific) for 5-HT or FMRF-amide. Following three 15-min washes in BBT, the larvae were stained with 4′,6-diamidino-2-phenylindole (DAPI) (Sigma, final concentration 2 µg/mL) and rhodamine-phalloidin (Invitrogen (Massachusetts), final concentration 1:40) for 1 h at room temperature to visualize the nuclei and F-actin, respectively. The stained specimens were washed for 15 min with PBT three times, mounted in VECTASHEILD mounting medium (Vector Laboratories, California) between coverslips supported with spacers, and then observed under a confocal laser scanning microscope FV3000 (Olympus, Tokyo).

Images were analyzed with Fiji in Image J ver. 1.53 and Photoshop 2021 (Adobe Inc., California), and figure plates made with Illustrator 2021 (Adobe Inc., California).

## Data Availability

The datasets used and/or analyzed during the current study are available from the corresponding author upon reasonable request. DNA sequences for the animals used in this study are available in GenBank under accession numbers: OR592590 and OR592591.
